# Supplementary material for: Integrating metabolic scaling and coexistence theories
Source: Ecology. 2025 Aug 5;106(8):e70173. doi: 10.1002/ecy.70173 (PMC12322717; doi:10.1002/ecy.70173)
Supplement: Supplementary file 1 — Appendix S1: [file ECY-106-e70173-s001.pdf]

Ecology
Appendix S1
Integrating metabolic scaling and coexistence theories

Serguei Saavedra, José Ignacio Arroyo, Jie Deng,
Pablo A. Marquet, Christopher P. Kempes

Derivation of competition interactions

The estimation of time-independent, area-independent, competitive interactions a_{ij} is derived by translating classic consumer-resource dynamics into Lotka-Volterra competition dynamics following Vandermeer and Goldberg (2013).

Let us assume that there are two self-replicating resources:

$$\begin{cases} \dfrac{\mathrm{d}R_1}{\mathrm{d}t} = r_{\mathrm{rsc}_1} R_1 \left[\left(\dfrac{k_1 - R_1}{k_1} \right) - c_{11}N_1 - c_{12}N_2 \right], \\ \dfrac{\mathrm{d}R_2}{\mathrm{d}t} = r_{\mathrm{rsc}_2} R_2 \left[\left(\dfrac{k_2 - R_2}{k_2} \right) - c_{21}N_1 - c_{22}N_2 \right], \end{cases} \tag{S1}$$

where R_i is the biomass density of the resource i (dimensions [mass]·[area]$^{-1}$), r_{rsc_i} is the intrinsic rate of increase of resource i (dimension [time]$^{-1}$), k_i is the carrying capacity of biomass density of the resource i (dimensions [mass]·[area]$^{-1}$), c_{ij} is the time-independent rate at which the resource i is consumed by the jth consumer across unit area (dimensions [mass$_j$]$^{-1}$·[area]), and N_i is the population biomass density of the ith consumer (dimensions [mass]·[area]$^{-1}$).

Then, considering two consumers, we can write the consumer equations:

$$\begin{cases} \dfrac{\mathrm{d}N_1}{\mathrm{d}t} = N_1 \left(b_{11}R_1 + b_{12}R_2 - d_1 \right), \\ \dfrac{\mathrm{d}N_2}{\mathrm{d}t} = N_2 \left(b_{21}R_1 + b_{22}R_2 - d_2 \right), \end{cases} \tag{S2}$$

where b_{ij} is the relative conversion of a unit of resource j by consumer i across unit area per time (dimensions [mass$_j$]$^{-1}$ · [time]$^{-1}$·[area]), and d_i is the death rate of the ith consumer (dimension [time]$^{-1}$). Note that b_{ij} is different from mass transformation efficiency.

Assuming that the dynamics of the resources occur significantly faster than those of the consumers (MacArthur and Levins, 1967), we can equate the derivatives of Eqs. (S1) to zero and solve for the equilibrium values R_1^* and R_2^* of the two resources:

$$\begin{aligned} R_1^* &= k_1(1 - c_{11}N_1 - c_{12}N_2), \\ R_2^* &= k_2(1 - c_{21}N_1 - c_{22}N_2). \end{aligned} \tag{S3}$$

Then, we can substitute R_1 and R_2 in Eqs. (S2) by R_1^* and R_2^*, respectively. That is,

$$\begin{cases} \dfrac{\mathrm{d}N_1}{\mathrm{d}t} = N_1 \left[b_{11}k_1(1 - c_{11}N_1 - c_{12}N_2) + b_{12}k_2(1 - c_{21}N_1 - c_{22}N_2) - d_1 \right], \\ \dfrac{\mathrm{d}N_2}{\mathrm{d}t} = N_2 \left[b_{21}k_1(1 - c_{11}N_1 - c_{12}N_2) + b_{22}k_2(1 - c_{21}N_1 - c_{22}N_2) - d_2 \right], \end{cases} \tag{S4}$$

which can also be written as

$$\begin{cases} \dfrac{\mathrm{d}N_1}{\mathrm{d}t} = N_1 \left[b_{11}k_1 + b_{12}k_2 - d_1 - (b_{11}k_1c_{11} + b_{12}k_2c_{21})N_1 - (b_{11}k_1c_{12} + b_{12}k_2c_{22})N_2 \right], \\ \dfrac{\mathrm{d}N_2}{\mathrm{d}t} = N_2 \left[b_{21}k_1 + b_{22}k_2 - d_2 - (b_{21}k_1c_{11} + b_{22}k_2c_{21})N_1 - (b_{21}k_1c_{12} + b_{22}k_2c_{22})N_2 \right]. \end{cases} \tag{S5}$$

Multiplying and dividing the two equations in Eqs. (S5) by

$$\begin{aligned} &b_{11}k_1 + b_{12}k_2 - d_1, \\ &b_{21}k_1 + b_{22}k_2 - d_2, \end{aligned} \tag{S6}$$

respectively, we obtain

$$\begin{cases} \dfrac{\mathrm{d}N_1}{\mathrm{d}t} = (b_{11}k_1 + b_{12}k_2 - d_1)N_1 \left[\dfrac{b_{11}k_1 + b_{12}k_2 - d_1 - (b_{11}k_1c_{11} + b_{12}k_2c_{21})N_1 - (b_{11}k_1c_{12} + b_{12}k_2c_{22})N_2}{b_{11}k_1 + b_{12}k_2 - d_1}\right], \\ \dfrac{\mathrm{d}N_2}{\mathrm{d}t} = (b_{21}k_1 + b_{22}k_2 - d_2)N_2 \left[\dfrac{b_{21}k_1 + b_{22}k_2 - d_2 - (b_{21}k_1c_{11} + b_{22}k_2c_{21})N_1 - (b_{21}k_1c_{12} + b_{22}k_2c_{22})N_2}{b_{21}k_1 + b_{22}k_2 - d_2}\right]. \end{cases} \tag{S7}$$

Again, dividing both the numerator and denominator of the fractions on the right sides of the two equations in Eqs. (S7) by

$$\begin{aligned} &b_{11}k_1c_{11} + b_{12}k_2c_{21}, \\ &b_{21}k_1c_{12} + b_{22}k_2c_{22}, \end{aligned} \tag{S8}$$

respectively, gives us

$$\begin{cases} \dfrac{\mathrm{d}N_1}{\mathrm{d}t} = (b_{11}k_1 + b_{12}k_2 - d_1)N_1 \left[\dfrac{\frac{b_{11}k_1+b_{12}k_2-d_1}{b_{11}k_1c_{11}+b_{12}k_2c_{21}} - N_1 - \frac{b_{11}k_1c_{12}+b_{12}k_2c_{22}}{b_{11}k_1c_{11}+b_{12}k_2c_{21}}N_2}{\frac{b_{11}k_1+b_{12}k_2-d_1}{b_{11}k_1c_{11}+b_{12}k_2c_{21}}}\right], \\ \dfrac{\mathrm{d}N_2}{\mathrm{d}t} = (b_{21}k_1 + b_{22}k_2 - d_2)N_2 \left[\dfrac{\frac{b_{21}k_1+b_{22}k_2-d_2}{b_{21}k_1c_{12}+b_{22}k_2c_{22}} - \frac{b_{21}k_1c_{11}+b_{22}k_2c_{21}}{b_{21}k_1c_{12}+b_{22}k_2c_{22}}N_1 - N_2}{\frac{b_{21}k_1+b_{22}k_2-d_2}{b_{21}k_1c_{12}+b_{22}k_2c_{22}}}\right]. \end{cases} \tag{S9}$$

Essentially, Eqs. (S9) are the Lotka-Volterra competition dynamics, which can be easily recognized if we make the following substitutions

$$\begin{aligned} r_1 &\triangleq b_{11}k_1 + b_{12}k_2 - d_1, \\ r_2 &\triangleq b_{21}k_1 + b_{22}k_2 - d_2, \\ K_1 &\triangleq \frac{b_{11}k_1 + b_{12}k_2 - d_1}{b_{11}k_1c_{11} + b_{12}k_2c_{21}}, \\ K_2 &\triangleq \frac{b_{21}k_1 + b_{22}k_2 - d_2}{b_{21}k_1c_{12} + b_{22}k_2c_{22}}, \end{aligned} \tag{S10}$$

and

$$\begin{aligned} a_{12} &\triangleq \frac{b_{11}k_1c_{12} + b_{12}k_2c_{22}}{b_{11}k_1c_{11} + b_{12}k_2c_{21}} = \frac{C_2(b_{11}k_1n_{12} + b_{12}k_2n_{22})}{C_1(b_{11}k_1n_{11} + b_{12}k_2n_{21})} = \frac{C_2}{C_1} \cdot \text{normalization constant}, \\ a_{21} &\triangleq \frac{b_{21}k_1c_{11} + b_{22}k_2c_{21}}{b_{21}k_1c_{12} + b_{22}k_2c_{22}} = \frac{C_1(b_{21}k_1n_{11} + b_{22}k_2n_{21})}{C_2(b_{21}k_1n_{12} + b_{22}k_2n_{22})} = \frac{C_1}{C_2} \cdot \text{normalization constant}, \end{aligned} \tag{S11}$$

where r_i is the rate of natural increase in isolation of consumer i (dimension $[\text{time}]^{-1}$), K_i is the carry capacity of consumer i biomass density in the absence of all competitors (dimensions $[\text{mass}]\cdot[\text{time}]^{-1}$), and a_{ij} is the time-independent, area-independent, competition coefficient measuring the competitive effect of consumer j on consumer i (dimensions $[\text{mass}_i]\cdot[\text{mass}_j]^{-1}$). Specifically, C_j is the time-independent average rate at which consumer j consumes resources across unit area (dimensions $[\text{mass}]^{-1}\cdot[\text{area}]$). Furthermore, it is typically observed that the relative conversion b_{ij} of a unit of resource j by consumer i and the carrying capacity k_j of resource j have limited variations for a given consumer i across different resources j and can be assumed constant for the sake of model simplification (Brown et al., 2004). Therefore, as indicated by Eq. (S11), it is important to note that the competition coefficient a_{ij} is proportional to the ratio of consumption rates C between consumer j and consumer i. Formally, we can represent it as

$$a_{ij} = a_0' \left(\frac{C_j}{C_i}\right), \tag{S12}$$

where a_0' is an *effective* parameter (dimensionless) representing the overall effect of intrinsic properties, such as consumers' conversion rates (Parain et al., 2019).

To further estimate the time-independent average rate C_i at which consumer i consumes resources, we can simplify the previous scenario by considering a streamlined framework that involves only one consumer (also predator, either consumer j or consumer i) and one resource (also prey). Specifically, if the resource is self-replicating, then the resource equation with a logistic growth is

$$\frac{\mathrm{d}R}{\mathrm{d}t} = rR\left[\left(\frac{k-R}{k}\right) - cN\right], \tag{S13}$$

where R is the biomass density of the resource (dimensions [mass]·[area]$^{-1}$), r is its per capita rate of increase (dimension [time]$^{-1}$), k is the carrying capacity of biomass density of the resource (dimensions [mass]·[area]$^{-1}$), c is the time-independent rate at which the resource is consumed by the consumer across unit area (dimensions [area]·[mass]$^{-1}$), and N is the biomass density of the consumer (dimensions [mass]·[area]$^{-1}$).

Then, we can write the consumer equation as

$$\frac{\mathrm{d}N}{\mathrm{d}t} = (bR - d)N, \tag{S14}$$

where b is the area-independent predation rate (dimensions [mass$_R$]$^{-1}$·[time]$^{-1}$) and d is the death rate of the consumer (dimension [time]$^{-1}$).

Assuming again that the resource dynamics are very fast compared to the consumer dynamics (MacArthur and Levins, 1967), we can solve the equilibrium value of R:

$$R^* = k(1 - CN), \tag{S15}$$

and then substitute R^* in place of R in the consumer equation Eq. (S14). We obtain

$$\frac{\mathrm{d}N}{\mathrm{d}t} = [bk(1 - CN) - d]N, \tag{S16}$$

which can also be written as

$$\frac{\mathrm{d}N}{\mathrm{d}t} = N(bk - d - bkCN). \tag{S17}$$

Multiplying and dividing Eq. (S17) by $(bk - m)$ gives us

$$\frac{\mathrm{d}N}{\mathrm{d}t} = (bk - d)N\left(\frac{bk - d - bkCN}{bk - d}\right). \tag{S18}$$

Next, dividing both the numerator and the denominator of the fraction on the right side of the Eq. (S18) by bkC, we have

$$\frac{\mathrm{d}N}{\mathrm{d}t} = (bk - m)N\left(\frac{\frac{bk-d}{bkC} - N}{\frac{bk-d}{bkC}}\right). \tag{S19}$$

If we denote

$$\begin{aligned} r &\triangleq bk - d, \\ K &\triangleq \frac{bk - d}{bkC}, \end{aligned} \tag{S20}$$

then Eq. (S19) can be written as the classic population model with logistic growth:

$$\frac{\mathrm{d}N}{\mathrm{d}t} = rN\left(\frac{K - N}{K}\right), \tag{S21}$$

where again r is the rate of natural increase in isolation of the consumer (dimension $[\text{time}]^{-1}$) and K is the carrying capacity of the consumer without competitors (dimensions $[\text{mass}]\cdot[\text{area}]^{-1}$).

Importantly, Eqs. (S20) indicate that the time-independent consumption rate C increases monotonically with the intrinsic growth rate r (Deng et al., 2024). Formally, by substituting the expression for bk as $(r + d)$ from the first equation into the second equation, we obtain

$$C = \frac{1}{K\left(1 + \frac{d}{r}\right)}. \tag{S22}$$

The time-independent consumption rate C (dimensions $[\text{area}]\cdot[\text{mass}]^{-1}$) exhibits a monotonically decreasing behavior with respect to the carrying capacity K (dimensions $[\text{mass}]\cdot[\text{area}]^{-1}$). Note that the relationship between carrying capacity K and intrinsic growth rate r appears to be complex and context-dependent (Marshall et al., 2023). Therefore, instead of establishing relationships between r and K, our primary focus lies on K due to its role in the feasibility of Lotka-Volterra competition dynamics. However, the focus can be shifted to r (Deng et al., 2024).

Following metabolic scaling theory (Eqs. 2-3 in main text), one can write the time-independent average consumption rate of population i (Eq. 35) as a function of body size (M_i). Note that mortality rate (d_i) is the reciprocal of generation time (G_i) and proportional to growth rate (r_i). For a fixed thermal domain and unique thermal response, we can replace the parameters in Eq. 37 with the following equivalences: $r_i = r_0 \cdot M_i^{-1/4}$, $d_i = d_0 \cdot M_i^{-1/4}$, $K_i = K_0 \cdot M_i^{-3/4} \cdot M_i = K_0 \cdot M_i^{1/4}$, where r_0, d_0, and K_0 are normalization constants. Thus, Eq. (35) can be expressed as:

$$C_i = a_0'' \cdot M_i^{-1/4}. \tag{S23}$$

Eventually, by combining Eq. (S12) with Eq. (36), we can derive an estimation of time-independent, area-independent, competitive interaction strength a_{ij} for a fixed thermal domain and unique thermal response as:

$$a_{ij} = a_0 \cdot (M_j/M_i)^{-1/4}, \tag{S24}$$

where a_0', a_0'', and a_0 are normalization constants. Based on this estimation, we can generate the competition matrix $\mathbf{A}$ for the Lotka-Volterra competition dynamics in Eq. (**??**) in main text.

Under $a_{ij} = a_0 \cdot (M_j/M_i)^{-1/4}$ (Eq. 39), Figure 2 (main text) shows that if the carrying capacities of biomass densities are defined as $K_i = K_0 \cdot M_i^{-3/4} \cdot M_i = K_0 \cdot M_i^{1/4}$, then they are the closest to the centroid of the feasibility domain. However, these carrying capacities $\boldsymbol{K}$ are expected to become exactly the centroid only under a specific value of a_0. For example, following Eq. 39 and for two competing species, the maximum and minimum feasible carrying capacities for species 2 (corresponding to the spanning vectors of the feasibility domain in Fig. 1) can be defined as $K_{2\max} = (a_{22}/a_{12})K_1$ and $K_{2\min} = (a_{21}/a_{11})K_1$, where $K_1 = K_0 \cdot M_1^{1/4}$ is the carrying capacity of biomass density of species 1 per metabolic scaling theory. Then, following Eq. 9, the carrying capacity for species 2 that belongs to the centroid of the feasibility domain becomes:

$$K_{2\text{c}} = \frac{1}{2}(K_{2\max} + K_{2\min}). \tag{S25}$$

Note that in the centroid the distance beteween K_{2c} and K_1 should be the absolute minimum, i.e., $K_{2c}/K_1 = 1$. Thus, dividing Eq. 40 by the carrying capacities of biomass density of species 1 (K_1) and rearranging it yields:

$$(M_2/M_1)^{-1/4} = \frac{1}{2} \cdot \frac{1 + a_0^2}{a_0} \cdot (M_2/M_1)^{-1/4}. \tag{S26}$$

This implies that the equality is satisfied if and only if the factor $\frac{1+a_0^2}{2a_0} = 1$. Since a_0 also affects the dynamical stability of the community, future work can investigate the possible trade-offs between stability and feasibility under this framework. However, under numerical simulations, the larger the community, the closer this expression becomes the centroid of the feasibility domain.

The vector $K_i = K_0 \cdot M_i^{1/4}$ tends to get asymptotically closer to the centroid of the feasibility domain the larger the community (Fig S1). Specifically, following Eq. 14 in main text, we generated an ensemble of 10^4 competition matrices $\mathbf{A}$ characterizing the time-independent, area-independent, competitive effects (a_{ij}) between $10, 20, 50, 100, 200, 500,$ and 1000 populations. Each matrix was formed by drawing M_i values independently from a lognormal distribution $LN(0, 2)$. These distributions can change without affecting the qualitative results. Following previous work (Bunin, 2017, Dougoud et al., 2018), we set the effective parameter of competitive effects to $a_0 = |\mathcal{S}|^{-1/2}$ for $i \neq j$, otherwise $a_0 = 1$. This assumption follows the rationale that empirical interactions tend to be weak, stabilizing communities (Gellner et al., 2023, McCann et al., 1998). The greater the value of a_0, the greater the overall competition, and the smaller the range of the feasibility domain. Then, we calculated the average distance between $\boldsymbol{K_c}(\mathbf{A})$ and $\boldsymbol{K}$ as (Medeiros et al., 2021, Saavedra et al., 2017)

$$d(\mathbf{A}, \boldsymbol{K}) = \arccos(\boldsymbol{K} \cdot \boldsymbol{K_c}(\mathbf{A}) \cdot ||\boldsymbol{K}||^{-1} \cdot ||\boldsymbol{K_c}(\mathbf{A})||^{-1}) \in [0, 90]. \tag{S27}$$

Note that the normalization constant K_0 of carrying capacities of biomass densities does not affect this distance and can be omitted from the equation (Rohr et al., 2016). This numerical analysis reveal that the large the number of populations, the smaller the distance to the centroid.

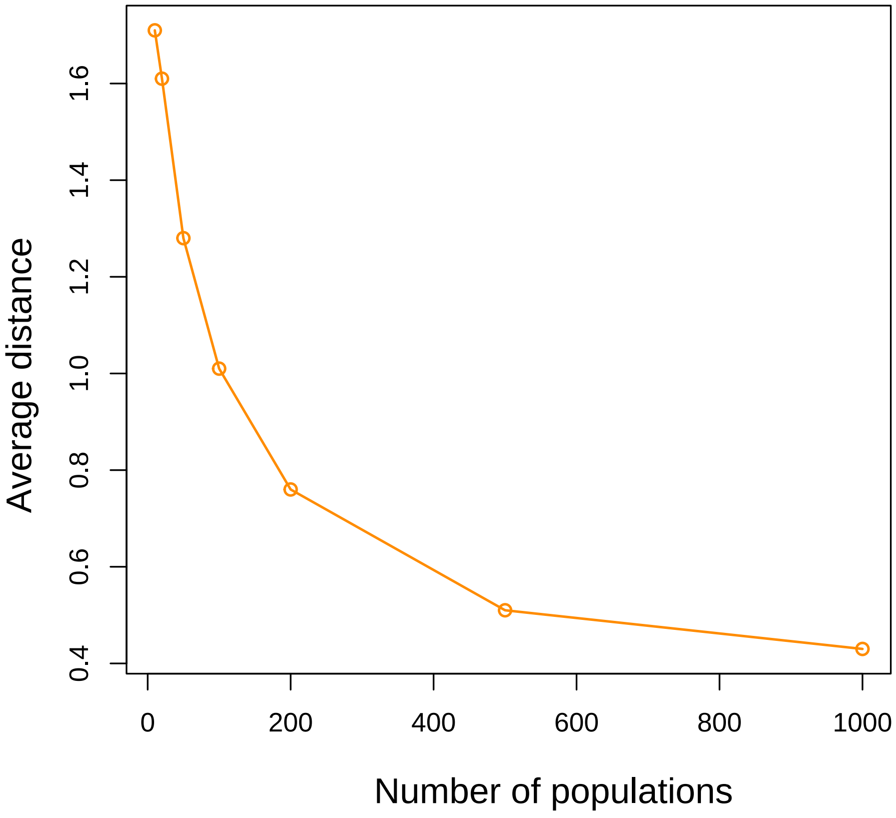

Figure S1: **Distance to the centroid for large communities**. For an ensemble of 10^4 generated competition matrices with different number of populations, we show the average distance between the vector $K_i = K_0 \cdot M_i^{1/4}$ and the centroid of the feasibility domain. This numerical analysis reveal that the large the number of populations, the smaller the distance to the centroid.

References

Brown, J. H., Gillooly, J. F., Allen, A. P., M., V., and West, G. B. 2004. Toward a metabolic theory of ecology. Ecology 85:1771–1789.

Bunin, G. 2017. Ecological communities with Lotka-Volterra dynamics. Physical Review E 95:042414.

Deng, J., Cordero, O. X., Fukami, T., Levin, S. A., Pringle, R. M., Solé, R., and Saavedra, S. 2024. The development of ecological systems along paths of least resistance. Current Biology 34:1–11.

Dougoud, M., Vinckenbosch, L., Rohr, R. P., Bersier, L. F., and Mazza, C. 2018. The feasibility of equilibiria in large ecosystems: a primary but neglected concept in the complexity-stability debate. PLOS Computational Biology 14:e1005988s.

Gellner, G., McCann, K., and Hastings, A. 2023. Stable diverse food webs become more common when interactions are more biologically constrained. PNAS 120:e2212061120.

MacArthur, R. and Levins, R. 1967. The limiting similarity, convergence, and divergence of coexisting species. Am. Nat. 101:377–385.

Marshall, D. J., Cameron, H. E., and Loreau, M. 2023. Relationships between intrinsic population growth rate, carrying capacity and metabolism in microbial populations. The ISME Journal Pages 1–4.

McCann, K., Hastings, A., and Huxel, G. R. 1998. Weak trophic interactions and the balance of nature. Nature 395:794–798.

Medeiros, L. P., Song, C., and Serguei, S. 2021. Merging dynamical and structural indicators of resilience. J. of Animal Ecology 90:2027–2040.

Parain, E. C., Rohr, R. P., Gray, S. M., and Bersier, L.-F. 2019. Increased temperature disrupts the biodiversity–ecosystem functioning relationship. The American Naturalist 193:227–239.

Rohr, R. P., Saavedra, S., Peralta, G., Frost, C. M., Bersier, L.-F., Bascompte, J., and Tylianakis, J. M. 2016. Persist or produce: a community trade-off tuned by species evenness. Am. Nat. 188:411–422.

Saavedra, S., Rohr, R. P., Bascompte, J., Godoy, O., Kraft, N. J., and Levine, J. M. 2017. A structural approach for understanding multispecies coexistence. Ecological Monographs 87:470–486.

Vandermeer, J. H. and Goldberg, D. E., 2013. Population ecology: first principles. Princeton University Press.
